# Cardio protective effect of nicorandil in reperfusion injury among patients undergoing primary percutaneous coronary intervention

**DOI:** 10.12669/pjms.39.1.6034

**Published:** 2023

**Authors:** Maria Ilyas, Mudassar Noor, Hamid Sharif Khan, Sauleha Haroon, Kulsoom Farhat, Shabana Ali

**Affiliations:** 1 Dr. Maria Ilyas, MBBS., Department of Pharmacology, Army Medical College, National University of Medical Sciences, Rawalpindi, Pakistan; 2 Dr. Mudassar Noor, Associate Professor of Pharmacology, Department of Pharmacology, Army Medical College, National University of Medical Sciences, Rawalpindi, Pakistan; 3 Dr. Hamid Sharif Khan, Assistant Professor of Cardiology, Department of Pharmacology, Army Medical College, National University of Medical Sciences, Rawalpindi, Pakistan, Rawalpindi Institute of Cardiology, Rawalpindi, Pakistan; 4 Dr. Sauleha Haroon, Senior Registrar of Cardiology, Department of Pharmacology, Army Medical College, National University of Medical Sciences, Rawalpindi, Pakistan, Rawalpindi Institute of Cardiology, Rawalpindi, Pakistan; 5 Dr. Kulsoom Farhat, Professor of Pharmacology, Department of Pharmacology, Army Medical College, National University of Medical Sciences, Rawalpindi, Pakistan; 6 Dr. Shabana Ali, Assistant Professor of Pharmacology, Department of Pharmacology, Army Medical College, National University of Medical Sciences, Rawalpindi, Pakistan

**Keywords:** Nicorandil, Percutaneous coronary intervention, myocardial reperfusion injury, Cardio protective agents

## Abstract

**Objectives:**

To evaluate the effect of nicorandil in prevention of reperfusion injury during primary percutaneous coronary intervention by thrombolysis in myocardial infarction flow grade scoring.

**Methods:**

A total of 140 patients from Rawalpindi Institute of Cardiology were enrolled in this study conducted from 7^th^ September to 10^th^ of October 2021. These participants were allocated into two major groups. Control group received conventional acute coronary syndrome protocol regimen only whereas experimental group was given nicorandil along with conventional acute coronary syndrome protocol. During primary percutaneous coronary intervention, thrombolysis in myocardial infarction flow grade scoring was analyzed and compared.

**Results:**

Majority of participants in nicorandil group achieved thrombolysis in myocardial infarction Grade-3 scoring which indicated reduced rate of no reflow phenomenon as compared to control group. A statistically significant difference was noted in score of both groups (*p* value = 0.001) signifying prophylactic use of nicorandil before primary percutaneous coronary intervention along with conventional acute coronary syndrome protocol is superior to only conventional acute coronary syndrome protocol regimen to cases in the control group.

**Conclusion:**

Use of nicorandil in ST elevated myocardial infarction patients before primary percutaneous coronary intervention prevents reperfusion injury thus decreasing the risk of post percutaneous coronary intervention complications and reducing mortality rate in cardiac patients suggesting its significant cardio protective role.

## INTRODUCTION

Approximately 17.9 million demises around the globe occur yearly mainly due to cardiovascular diseases (CVDs) and this number is expected to increase drastically in the future overall increasing health burden on all national and international health care systems.^1^,^2^ Coronary heart disease (CHD) is the term mainly reserved for the narrowing of vessels supplying major oxygenated blood to the cardiac tissue. Ischemic changes in any area are mainly triggered by the obstruction of blood vessel either mechanically or physically leading to apoptosis or necrosis of that specific area.^3^

Myocardial infarction (MI) occurs when any epicardial coronary artery is abruptly occluded by the rupture of atherosclerotic plaque. It is one of the foremost causes of death worldwide accounting for more than one million hospital admissions annually.^4^ It affects around 126 million people in the whole world which mark about 1.72% of the current world’s population. Nine million deaths were reported by ischemic heart diseases (IHD) globally affecting men more than women. Current prevalence rate of 1655 per 100,000 populations is expected to increase to 1845 by the year 2030.^5^ In Pakistan, CVDs are responsible for 29% of mortality rate. Atherosclerosis is mainly the disease of arterial wall comprising of several risk factors, some of which are modifiable and some are non-modifiable.^6^

Ischemic reperfusion (IR) injury occurs when poor blood supply of an ischemic tissue is improved via stenting or ballooning, ultimately reestablishing perfusion and oxygenation after a certain critical time period. This phenomenon mainly involves changes at cellular and molecular level like “mitochondrial permeability transition pore (MPTP)” opening is one of the mechanism behind this injury. After an ischemic attack, reperfusion is the standard treatment either by using medicines or interventional procedures. However, it can paradoxically cause cardiac dysfunction accounting for almost 10% to 40% cases of reperfusion injury and no reflow phenomenon (NRP).^7^ The pathological basis of IR injury is multifactorial and it involves variety of mechanisms at cellular, molecular and tissue level.^8^ NRP is defined when incomplete reperfusion of myocardium is achieved in the absence of any physical obstruction of coronary artery which can be easily seen through angiography. The prevalence rate of NRP in STEMI patients who underwent primary PCI is >30% and can cause adverse clinical outcome, including “arrhythmias, heart failure, sudden cardiac death” and other major cardiovascular complications.^9^,^10^

The treatment of MI involves both pharmacological and non-pharmacological approaches. Among the non-pharmacological treatment, primary PCI is the gold standard treatment used in STEMI patients.^11^ Timely myocardial reperfusion by this procedure has become an important treatment strategy which can reduce size of infarction, preserve cardiac muscle function and improve clinical outcome in these patients. However, despite effective reperfusion by PCI, a considerable percentage of patients with STEMI still have compromised cardiac function and it has been demonstrated that coronary micro vascular dysfunction is important cause of subsequent deterioration and bad prognosis of patients.^12^ This paradoxical outcome is mainly attributed to phenomenon known as IR injury and it leads to NRP. It may develop in 5–50% of STEMI patients during primary PCI.^13^

NRP has been recognized as one of the serious complications of PCI procedure mainly measured via scoring system called as TIMI (Thrombolysis in myocardial infarction) score.^14^,^15^ Nicorandil, with its novel mechanism of ischemic preconditioning (IP), by which it causes stabilization of inner mitochondrial membrane during reperfusion thus prevents opening MPTP and reducing apoptosis cardiac cells, in addition to its antianginal role via coronary vasodilatation. Several medicines are now being used to prevent reperfusion related complications and NRP like sodium nitroprusside, statins, adenosine and verapamil addressing different mechanisms of reperfusion injury and preventing them.^16^

Nicorandil is hybrid of an adenosine triphosphate (ATP) sensitive potassium channel opener and nitrates, has been proved to improve coronary micro vascular dysfunction via its vasodilatory effect and preventing reperfusion injury by mimicking action of IP. It is being used as a treatment modality of chronic stable angina and is also well tolerated with a suitable safety profile as evidenced by many meta-analysis reports. In addition to its benefits on coronary microcirculation, Nicorandil may also exert cardio-protective effect via its anti-oxidant and anti-inflammatory role.^17^

Our objective was to evaluate the effect of nicorandil in prevention of reperfusion injury during primary percutaneous coronary intervention by thrombolysis in myocardial infarction flow grade scoring.

## METHODS

This comparative analytical study was conducted at Rawalpindi Institute of Cardiology from 7th September to 10th October 2021. Sample size of 140 was calculated with the response rate of 90% by using Raosoft, an online sample size calculator. The participants were enrolled via non probability convenience sampling and they were divided into two groups A and B. Informed consent was taken from individuals before enrollment in this study and all of their demographic details were noted. Among the two groups, group A (n=70) received conventional ACS protocol which included tablet nitroglycerin 0.5 mg sublingually stat, tablet aspirin 300 mg stat, tablet clopidogrel 300 mg stat and injection heparin 5000 units intravenous whereas group B (n=70) was given Nicorandil along with the conventional ACS protocol. The subjects of both groups then underwent interventional procedure of primary PCI in cardiac catheterization laboratory. NRP was then measured by using TIMI flow grade scoring in both the groups. Reperfusion injury is defined as achieving less than Grade-3 TIMI flow grade scoring.^18^ “Flow in coronary arteries is classified as Grade-O no flow, Grade-1 penetration without perfusion, Grade-2 partial perfusion and Grade-3 complete perfusion”. TIMI grade 3 flow requires that ante grade flow distally be as rapid as ante grade flow proximally.^15^ Data was analyzed by using Statistical Package for Social Science (SPSS) version 26.0. Descriptive statistics were applied to analyze the frequencies and chi square test was used to highlight the statistical difference between both groups.

### Inclusion and Exclusion Criteria:

Patients qualified for this study were both male and female, aged 40 to 60 years, who presented with STEMI and ejection fraction more than 35% on echocardiography. The participants taking glibenclamide and glimipiride, with severe left ventricular dysfunction or those who already have received streptokinase before reaching hospital were excluded.

### Ethical Approval:

It was taken from Ethics Review Committee on 3^rd^ September 2021, Army Medical College, National University of Medical Sciences, Rawalpindi (ERC/ID/124) and Institutional Review Board of Rawalpindi Institute of Cardiology. A verbal and written consent was also taken by the recruited participants.

## RESULTS

Baseline parameters including age, gender, diabetes, hypertension, smoking, body mass index (BMI), triglycerides (TAG) and total cholesterol were statistically comparable (Table-I).

**Table-I:** Baseline parameters in both study groups.

Parameters		Control Group	Nicorandil Group	p Value
Age	40-50	25(35.07%)	18(25.70%)	0.20
	51-60	45(64.03%)	52(74.30%)	0.07
Gender	Male	58(82.09%)	65(92.90%)	0.59
	Female	12(17.01%)	5(7.1%)	0.61
Diabetes		46(65.1%)	49(70.0%)	0.59
Hypertension		29(41.4%)	32(45.7%)	0.61
Smoking		42(60.0%)	47(67.01%)	0.38
BMI		25.45±3.76	25.68±4.39	0.74
TAG		107.03±76.11	101.17±50.28	0.59
Total Cholesterol		173.90±37.08	171.00±36.82	0.64

**Fig.1:**
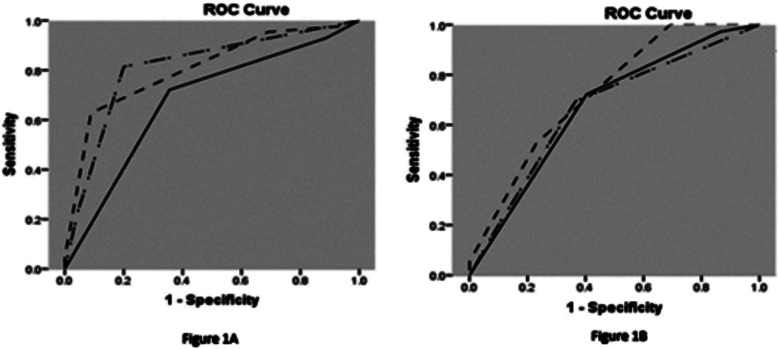


According to TIMI flow grade scoring, 14 (20.0%) cases in control group showed complete perfusion after PCI and 54 (77.1%) patients had partial perfusion. In Nicorandil group 61 (87.1%) cases had complete perfusion whereas 9 (12.9%) participants achieved partial perfusion. It implies that in comparison to control group, the rate of NRP after PCI was significantly lower in Nicorandil group (p value = 0.001) (Table-II and Fig.1).

**Table-II:** Distribution of TIMI flow grade scoring in both study groups.

TIMI Flow Grade Scoring	Study Groups		

	Nicorandil	Control	p Value
1	0(0.0%)	2(2.9%)	0.001
2	9(12.9%)	54(77.1%)	
3	61(87.1%)	14(20.0%)	

Total	70(100%)	70(100%)

## DISCUSSION

Numerous studies have been conducted to evaluate the effect of medicines on ischemic heart tissue and its reperfusion complications using different parameters including cardiac biomarkers, ECG changes, TIMI scoring and major cardiovascular events (MACE).^19^-^22^ In our study, we evaluated the cardio protective role of nicorandil in patients of STEMI who had undergone primary PCI. Nicorandil is being used clinically for treating chronic stable angina patients but its new mechanism of IP makes its role valuable in interventional procedure of PPCI. MPTP structure present on inner mitochondrial membrane opens up during reperfusion in response to various trigger factors like calcium overload and oxidative stress, this drug controls these factors as well as prevents MPTP opening directly thus reducing reperfusion injury chances.^17^ The patients were given nicorandil 20 mg orally stat along with conventional ACS protocol almost 60 to 90 minutes (door to balloon must <90 minutes) before PCI. TIMI flow grade scoring was assessed after PCI and it proved that nicorandil significantly reduced rate of NRP in patients who had taken the drug showing its phenomenal effects on ischemic heart tissue as compared to the control group. Similar studies had been carried out throughout the years to demonstrate its remarkable role in cardiac patients.

In the beginning when nicorandil use was in revolution phase, a study was done between 1998 and 2003 which continued for five years and enrolled 368 patients of STEMI who received 12 mg Nicorandil intravenously. After the procedure, 89.7% of cases had TIMI-3 grade.^21^ The results of this study matched with our study. It led to numerous studies on Nicorandil use other than as an anti-anginal drug in cardiac patients.

Another study was conducted on 19 patients in 2001. They administered sodium nitroprusside directly intracoronary via guiding catheter in a median dose of 200µg per patient. It was assessed that sodium nitroprusside directly released nitric oxide causing coronary vasodilatation and improved impaired blood flow. In the end, when results were analyzed and comprehended, there was significant improvement in NRP and TIMI flow grade scoring.^23^ These results were comparable to our present study findings adding weight to innovative use of nicorandil in the field of cardiology. Another subsequent study was conducted between 2011 and 2014 and it had comparable results with the current research. It involved 115 patients, divided into four groups. One group had nicorandil 2mg intracoronary before primary PCI, second group received 2mg anisodamine and third group had both Nicorandil plus anisodamine while fourth group was considered as control. TIMI score was assessed and it was improved in all groups as compared to control group but was markedly upgraded in last group in which two drugs were used in combination.^24^

A study conducted in India also revealed comparable results to our findings as they used nicorandil infusion two hours before PCI and continued for 48 hours after PCI and results were compared on basis of TIMI scoring and other angiographic parameters with control group and it had trend toward improvement in NRP but it could not reach statistical significance because of small sample size of only 42 patients.^25^

Intravenous nicorandil caused relative significant hypotension. To prevent this fatal outcome in PCI, researchers started using this drug orally side by side which led to many significant studies and their results. A project was carried out in China in 2015 by Yang and its colleagues which revealed that when different single oral doses (10mg and 20 mg) were given before PCI, cardiac markers’ levels reduced significantly 20 to 24 hours after PCI. TIMI flow grade scoring and ST segment elevation improved as compared to patients who received only conventional treatment. These single oral doses did not cause any hypotensive episode in participants proving equal efficacy of oral and intravenous nicorandil in cardio-protection.^20^ Oral nicorandil is as effective as parenteral because it lacks hepatic metabolism and has bioavailability of 75% to 80% comparable with intravenous or intracoronary route (100%). Peak plasma concentrations are also achieved in 0.5 to one hour (both in 5mg and even in 20 mg doses).^21^

Our study used single stat dose of nicorandil 20 mg before primary PCI and the results were comparable with the study of Pang et al.^19^ 2015, they compared use of Nicorandil given in dose of 10 mg three times a day for three days before elective PCI with control group which only received conventional therapy. Study analyzed cardiac parameters including TIMI scoring. Results showed less NRP in nicorandil group as compared to control group and all other parameters were also improved in experiment group confirming its cardio protective mechanism.^20^

The most reliable explanation of beneficial effect of cardio protection achieved by oral nicorandil is that it mimics IP and prevents reperfusion injury by acting at mitochondrial level causing stabilization of inner mitochondrial membrane causing decrease in NRP incidence. Our present study results correlate with all the previous studies conducted on nicorandil in the last years and proving its use as prophylactic drug to prevent reperfusion injury if given before intervention.

### Limitations:

Firstly, it had relatively small sample size which can be further increased. Secondly, it was a single center study with not much ethnic variation available therefore multicenter study can be performed and results can be applied to large number of population. Thirdly, prolonged follow up of participants should be done and see the long term beneficial results of this drug on ischemic heart tissue. If these limitations are addressed, significance of study can be significantly enhanced.

## CONCLUSION

The use of Nicorandil before primary PCI in STEMI patients effectively reduces chances of perioperative myocardial injury and NRP. It can be used safely as prophylactic drug before interventional procedure and reduces incidence of reperfusion injury in cardiac tissue which is assessed by TIMI flow grade scoring thus preventing myocardial stunning and improving myocardial perfusion status and ultimately cardiac function.

### Authors’ Contribution:

**MI:** Conceived, designed, collected the data, did statistical analysis with interpretation & manuscript writing.

**MN:** Supervised the entire project right from its conception and critically revised the article for important intellectual content.

**HS & SH:** Supervised the entire project in the clinical setting and revised the manuscript critically for final publication.

**KF & SA:** Did review and final approval of manuscript, along with ensuring accuracy and integrity of entire data.
